# SEM Analysis of the Interfacial Transition Zone between Cement-Glass Powder Paste and Aggregate of Mortar under Microwave Curing

**DOI:** 10.3390/ma9090733

**Published:** 2016-08-27

**Authors:** Yaning Kong, Peiming Wang, Shuhua Liu, Guorong Zhao, Yu Peng

**Affiliations:** 1 School of Materials Science and Engineering, Tongji University, Shanghai 201804, China; kongyaning1224@126.com (Y.K.); zhaoguorong1986@163.com (G.Z.); 2 State Key Laboratory of Water Resources and Hydropower Engineering Science, Wuhan University, Wuhan 430072, China; shliu@whu.edu.cn; 3 College of Civil Engineering and Architecture, Zhejiang University, Hangzhou 310000, China; pengyucs@gmail.com

**Keywords:** SEM, microstructure, ITZ, microwave curing, glass powder

## Abstract

In order to investigate the effects of microwave curing on the microstructure of the interfacial transition zone of mortar prepared with a composite binder containing glass powder and to explain the mechanism of microwave curing on the improvement of compressive strength, in this study, the compressive strength of mortar under microwave curing was compared against mortar cured using (a) normal curing at 20 ± 1 °C with relative humidity (RH) > 90%; (b) steam curing at 40 °C for 10 h; and (c) steam curing at 80 °C for 4 h. The microstructure of the interfacial transition zone of mortar under the four curing regimes was analyzed by Scanning electron microscopy (SEM). The results showed that the improvement of the compressive strength of mortar under microwave curing can be attributed to the amelioration of the microstructure of the interfacial transition zone. The hydration degree of cement is accelerated by the thermal effect of microwave curing and Na^+^ partially dissolved from the fine glass powder to form more reticular calcium silicate hydrate, which connects the aggregate, calcium hydroxide, and non-hydrated cement and glass powder into a denser integral structure. In addition, a more stable triangular structure of calcium hydroxide contributes to the improvement of compressive strength.

## 1. Introduction

Curing period reduction of precast concrete production has many advantages, e.g., reduction of workshop area, capital saving, and productivity improvement. Steam curing, which takes at least 10 h to finish one curing cycle before demolding, is the most popular method for precast concrete production [1]. But concrete with low heat conductivity is apt to form a thermal gradient under steam curing, leading to temperature cracks formed by heat stress. High-frequency electromagnetic heating, such as microwave curing, is able to reduce such nonuniformity due to its superior penetration depth. In addition, microwave curing exhibits the characteristics of energy saving, rapid stripping for precast concrete [2,3] or concrete repair [4], which is effective in accelerating the hydration of cement [5,6,7,8] or improving the pozzolanic reaction of supplementary cementitious materials (SCMs) [9,10,11,12,13,14]. According to the research of Wu et al., microwave curing increases the early strength without any detrimental effect at later ages [15]. The specimens under microwave curing decreased the porosity, which is beneficial to the reduction of plastic shrinkage. Leung et al. also showed that early strength at 4.5 h and later age strength at 7 days of concrete prepared with type III Portland cement under microwave curing are very comparable with concrete containing accelerating admixtures as well as commercially available rapid hardening concrete [16]. Recently published work by Makul et al. showed that the strength of specimens under a Cober Electronics industrial microwave generator (2.45 GHz, 6.0 kW) increased when compared with that cured under autoclaved and lime saturated conditions, particularly at early ages [17].

Concrete consists of matrix, aggregate and interfacial transition zones (ITZ) [18]. ITZ is considered the weakest part of the concrete, leading to lower compressive strength than aggregate and hardened cement paste [19]. Furthermore, the micro cracks in ITZ contribute to the transformation of cement-based materials under loading from linear response to the nonlinear response [20]. From the mechanical perspective, the level of micro cracks in ITZ is determined by the mismatch degree of strain between aggregate and paste [21]. Under the condition of homogeneous stress, the difference of strain is attributed to the elasticity modulus and the shear modulus of aggregate, matrix, and ITZ and stress concentration. The ITZ bridges the matrix and aggregate. If the ITZ fails to transfer the stress from one phase to another, the compressive strength of concrete is weakened even with the phase having a higher stiffness [22]. In order to understand the mechanism of ITZ formation and the effects of ITZ on the concrete properties, microstructural analysis was performed in detail. It has been identified that enrichment of calcium hydroxide (Ca(OH)_2_, CH) and relatively high porosity and large capillary pores affect the mechanical properties significantly. The micro cracks are apt to form perpendicular to the c-axis because of the enrichment of CH around the aggregate and the c-axis orientation paralleling the surface of aggregate [18]. In addition, the large sized CH weakens the van der Waals force, resulting in the decrease of cementitiousness, which is adverse to the mechanical properties. In addition to the CH, micro cracks and high porosity are easy to form in ITZ, which reduce the mechanical properties further. In order to ameliorate the ITZ, supplementary cementitious materials (SCMs) are commonly used. SCMs can improve the mechanical property and durability and also improve the compactness and reduce the thickness of ITZ [23,24]. In this study, waste glass powder was used as SCM to improve the ITZ. Waste glass consists of mainly amorphous SiO_2_ (about 70%). The amorphous SiO_2_ can be dissolved in the alkali environment and react with calcium hydroxide to form secondary calcium silicate hydrate (C-S-H). In addition, a certain amount of CaO, Al_2_O_3_ and Na_2_O exists in glass [25,26]. Three reasons contribute to the accelerated hydration of cement at early ages. Firstly, the reaction of glass powder with Ca^2+^ in the solution; secondly, the catalytic action of Na^+^ dissolved from glass powder; and thirdly, the increase of sites for nucleation and growth of C-S-H by glass powder [27]. The secondary reaction activation of glass powder is mainly controlled by the fineness and the curing temperature. Increase of specific surface area [28] or increase of curing temperature [29] can improve the activation of glass powder. The pozzolanic reactivity of glass powder can improve the microstructure of paste, thereby ameliorating the strength and durability at later ages [30]. In addition, alkali-silica reaction expansion of mortar containing glass powder is negligible. Waste glass powder as a kind of SCM is also beneficial in the reduction of expansions in a mortar undergoing alkali-silica reaction due to the increased dissolution of Na^+^ by the glass waste aggregate [31,32,33].

In this study, the effects of microwave curing on the microstructure of the interfacial transition zone of mortar and the mechanism of microwave curing on the improvement of compressive strength were investigated. The compressive strength of mortar under microwave curing was compared with that cured using (a) normal curing at 20 ± 1 °C with RH > 90%; (b) steam curing at 40 °C for 10 h; and (c) steam curing at 80 °C for 4 h. The microstructure of the interfacial transition zone of mortar under the four curing regimes was analyzed by scanning electron microscopy/energy dispersive spectrometry (SEM-EDS).

## 2. Results

### 2.1. Compressive Strength

**Figure 1:**
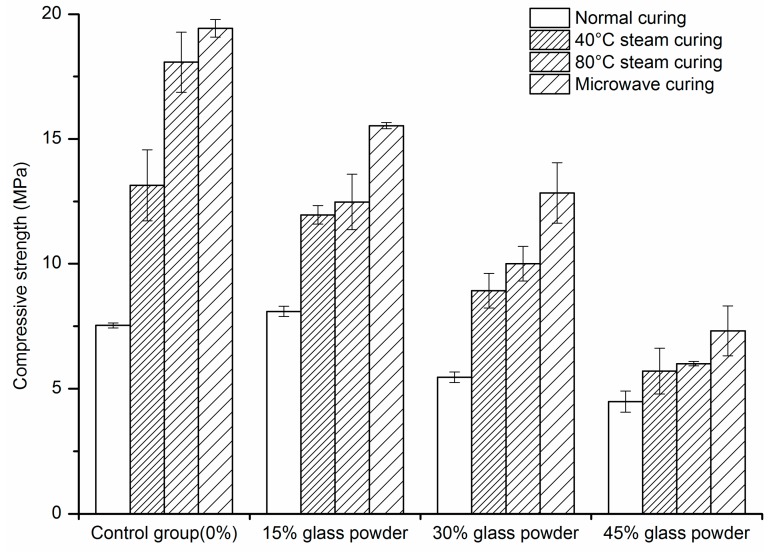
Compressive strength of mortar at one day.

**Figure 2:**
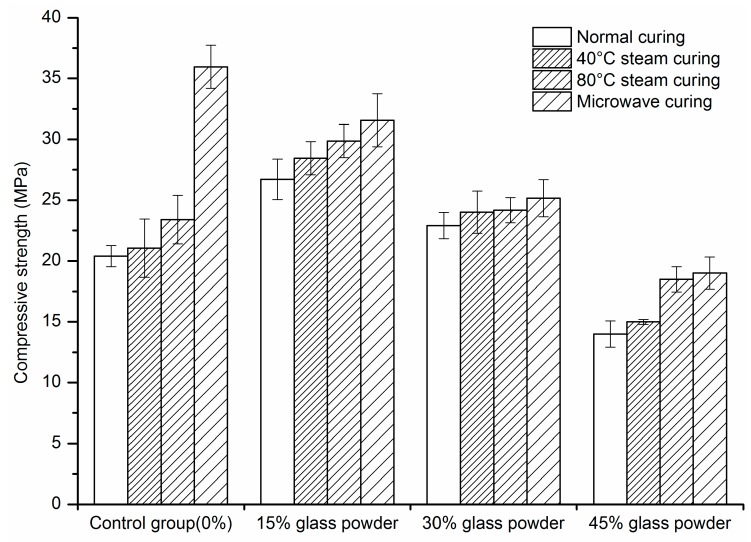
The compressive strength of mortar at 28 days.

The compressive strength of mortar at the age of one day and 28 days is shown in Figure 1 and Figure 2. At the age of one day, under normal curing, the compressive strength of mortar at the replacement ratio of 15% is slightly higher than the controls, which is attributed to the filling and nucleation effect of glass powder as SCM. Under steam curing, although the hydration of glass powder will be accelerated, the hydration of glass powder is much slower than that of the cement. Therefore, the compressive strength decreases with the increase of replacement ratio. The compressive strength of mortar under microwave curing is the highest among the four curing regimes. At the replacement ratio of 15% and 30%, the advantage of microwave curing is even more significant. This may be caused by a denser structure resulting from the filling effect, the nucleation effect of glass powder, and the plastic shrinkage of mortar by the decrease of water to binder ratio during the curing.

At the age of 28 days, with the replacement ratio of 15% or 30%, the pozzolanic reaction of glass powder under normal curing and steam curing becomes visible, leading to a compressive strength that is higher than the controls. However, at the replacement ratio of 45%, the compressive strength decreased, which is different than the research by Du et al. [34]. This is caused by the relatively small specific surface area. In this study, the specific surface area of glass powder was 270 m^2^/kg, which decreased its pozzolanic activity.

### 2.2. SEM Microanalysis

**Figure 3:**
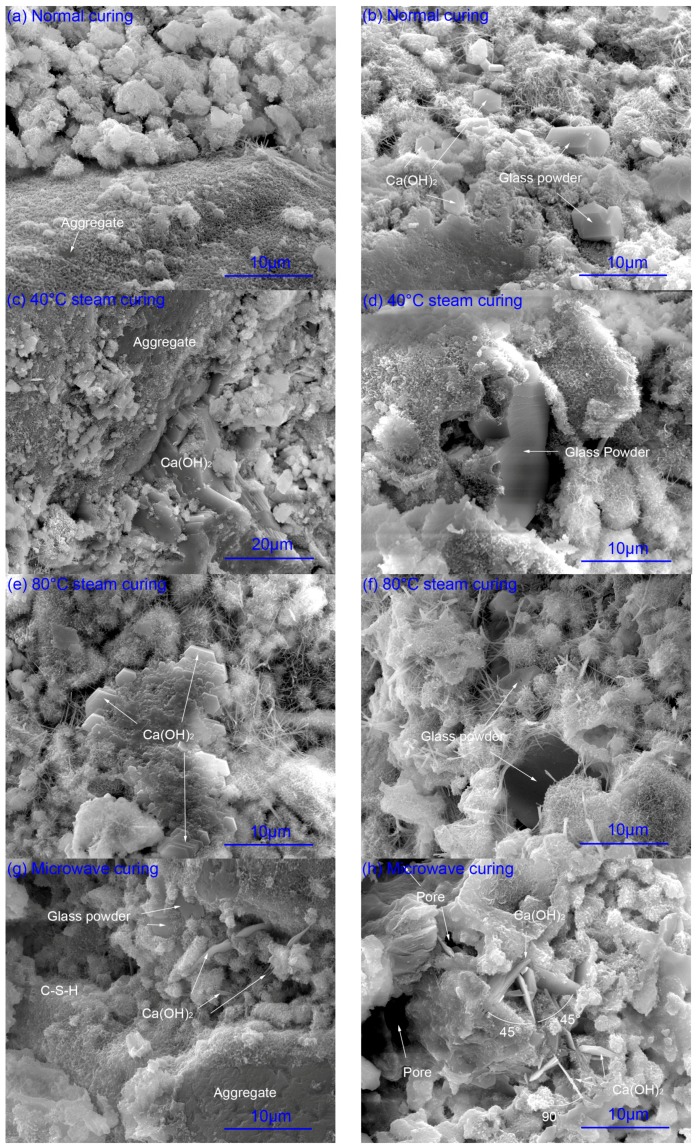

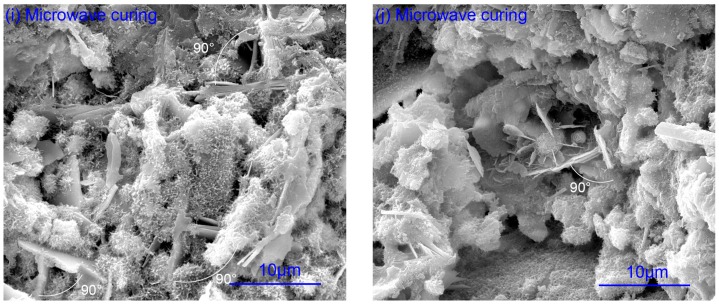
Fracture morphology of mortar prepared with composite binder containing 30 wt % glass powder at one day. (**a**,**b**) normal curing; (**c**,**d**) 40 °C steam curing; (**e**,**f**) 80 °C steam curing; (**g**–**j**) microwave curing.

**Figure 4:**
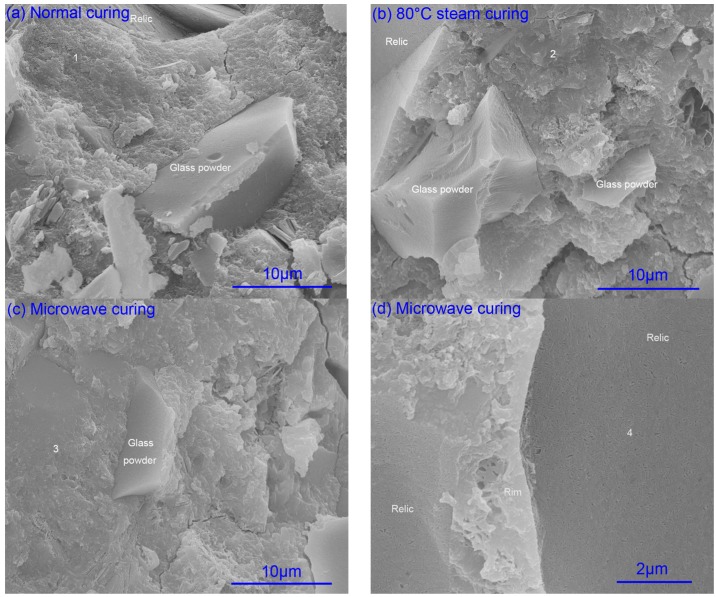
Fracture morphology of mortar at 28 days. (**a**) normal curing; (**b**) 80 °C steam curing; (**c**,**d**) microwave curing.

In order to investigate the effects of microwave curing on the ITZ, the fracture morphology of mortar prepared with composite binder containing 30% glass powder at the age of one day and 28 days was observed by SEM. The results are shown in Figure 3 and Figure 4. The element composition of hydration products were also analyzed by EDS, as listed in Table 1.

#### 2.2.1. Hydration of Glass Powder

At the age of one day, the surface of glass powder under normal curing (Figure 3a,b) is smooth, showing that the glass powder mainly plays a filling effect. The glass powder fills into the capillary pores and is locked mechanically with other phases to contribute to the compressive strength. Large amounts of C-S-H are precipitated on the surface of glass powder under steam curing (Figure 3d,f) and microwave curing (Figure 3g). Furthermore, the glass powder is wrapped into the dense C-S-H gel. The hydration of glass powder under microwave curing can also be reflected by the pH in Figure 7. The reaction of SiO_2_ in glass powder with calcium hydroxide hydrated by cement forms C-S-H gel and leads to the decrease of the pH at 6 h. Then, the hydration rate of cement is faster than that of glass powder, leading to an increase of pH after 6 h under microwave curing. According to the research of M. Mirzahosseini et al., the dissolution of Na^+^ increases significantly when the pH is higher than 13.5 with the temperature at 50 °C [25]. In this study, the content of Na_2_O is 13.2%. It can be seen from the Materials and Methods section that 42.7% of glass powder is smaller than 25 μm, which is conducive to the dissolution of Na [25]. Increase of Na^+^ in the solution can accelerate the hydration of cement [35,36]. The dissolved Na^+^ enters into the C-S-H around the glass powder to form alkali-silica reaction (ASR) gel with a high Na/Si atomic ratio as shown in Table 1, which is in agreement with the findings reported by Serpa et al. and Redden et al. [26,37].

#### 2.2.2. Calcium Hydroxide

The hydration degree of cement is the lowest under normal curing. Small sized CH with hexagonal-platelet morphology was observed in Figure 3a,b. Lamellar structure of CH under steam curing was formed (Figure 3c,e). As usual, orientation of CH around aggregate was observed. The connection of layers is by hydrogen bond, which is very weak. The structure and shape of CH indicates that CH rarely contributes to the compressive strength. The weak connection of layers of CH may be the headstream of micro cracks [38]. However, it can be seen from the Figure 3h–j that a 45° angle or 90° angle was formed under microwave curing between the layers of CH. The gap between the layers was filled with the precipitation of C-S-H. The CH layers and the C-S-H form a more stable triangular structure. The connection of CH and C-S-H either by mechanical lock or chemical bonding is stronger than the hydrogen bond between the layers of CH. This has a very weak adverse effect of CH on the compressive strength.

#### 2.2.3. Pore Structure

From the Figure 3a,b, the microstructure of ITZ of mortar under normal curing is very loose. Phases are locked mechanically. This is mainly attributed to the low hydration degree of cement. Steam curing increases the hydration extent, but there are still a lot of non-hydrated cement particles. The capillary pores between the cement particles are still obvious. In addition, some slender ettringite were formed under normal curing and steam curing in the pores (Figure 3b,d–f).

It can be seen from Figure 3h–j, more hydration products were formed under microwave curing. However, a lot of channels are observed. Microwave radiation can heat the mortar to 100 °C in half an hour or even a few minutes. The gasification of water and expansion of air in the mortar during the plastic stage will increase the capillary pores. The matrix cannot shrink after the microwave curing because of the hardening. In addition, the evaporation of water during microwave curing will also increase the channels during the water transport. From the morphology of the mortar under microwave curing, the diameter of the channels can be classified as capillary pores, which is adverse to the compressive strength and permeability [18]. However, as seen in Figure 3g, the aggregate, glass powder, and cement particles are connected by large amounts of reticular C-S-H into a whole to improve the compressive strength.

#### 2.2.4. Element Composition

Ca, Na, and the SiO_2_ network will be dissolved from the glass surface when the OH^−^ attacks the glass to form ASR gel as shown by Equations (1) and (2) [39,40]. In Figure 4d, the gel in the relic of glass powder can be defined as ASR gel according to the element analysis in Table 1.

(1) $${\text{Ca}}^{2+}+{\text{Na}}^{+}+{\left[\text{SiO}{\left(\text{OH}\right)}_{3}\right]}^{-}\to C-N-S-H$$

(2) $${\text{SiO}}_{2}+2{\text{Na}}^{+}\left(K^{+}\right)+2{\text{OH}}^{-}\to {\text{Na}}_{2}\left(K_{2}\right){\text{SiO}}_{3}\cdot H_{2}O$$

According to Table 1, microwave increases the Na/Si atom ratio, leading to the formation of C-S-H containing Na (C-N-S-H). The adsorption of Na^+^ caused by the secondary reaction of glass powder with CH makes the Na^+^ unavailable for reaction with silicate [41]. CaO/(SiO_2_ + Na_2_O) determines the swelling property of the ASR gel [42,43]. The relative high Ca^2+^, regardless of form, is beneficial to hindrance of ASR expansion. Therefore, the expansion caused by ASR may be restrained by microwave curing when compared with steam curing and normal curing.

## 3. Discussion

The hydration mechanism of a composite binder under microwave curing is similar to that under steam curing. The hydration of cement can be accelerated by thermal effects. The glass powder mainly plays the role of an inert filler at the early ages, which reduces the porosity of ITZ. At the same time, the partial dissolution of fine glass powder can be accelerated by the increase of temperature. The SiO_2_ dissolved from glass powder reacts with CH hydrated by cement to form C-S-H gel. The dissolved Na^+^ accelerates the hydration of cement to form C-S-H with a high Na/Si atomic ratio at the same time.

However, there is a significant difference of microstructure of ITZ between microwave curing and steam curing. In the mortar, except for the aggregate, all the phases contain dielectric materials. The dielectric properties of the materials are different because of relaxation and polarization. Ions will move quickly along with the alternating electromagnetic field. Water molecules are the easiest to influence by microwaves. The fracture of the hydrogen bond can be reflected by the surface tension. According to the results shown in Figure 8, microwaves can break the hydrogen bond to form OH^−^. OH^−^ will attack the Si-O bond to accelerate the hydration of cement and the dissolution of glass powder. In addition, the movement of ions, especially the Ca^2+^, with the microwave increases the probability of nucleation, which is good for the precipitation, i.e., C-S-H and CH formation. However, the hydrogen bond between the layers of CH is also broken by microwaves. The orientation of CH is also disturbed, resulting in a 45° or 90° angle between the layers of CH. The CH is then wrapped by C-S-H precipitation to form a more stable triangular structure. The triangular structure can act as fibers to hinder the micro crack propagation by cutting down the spread route. However, the air and water expands or gasifies rapidly during microwave radiation, resulting in the increase of capillary pores, which may be good for frost resistance but is adverse to the compressive strength. In a general, more reticular C-S-H connect the aggregate, glass powder, and non-hydrated cement particles into a denser integral structure. Therefore, although only 45 min of microwave radiation was performed, the compressive strength of mortar under microwave curing was improved significantly.

## 4. Materials and Methods

**Figure 5:**
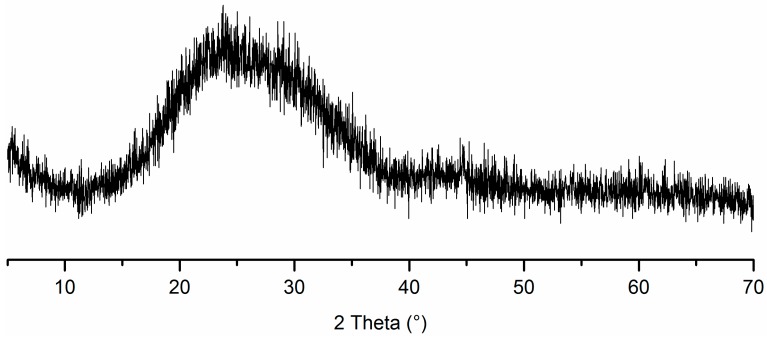
X-ray diffraction pattern of glass powder.

**Figure 6:**
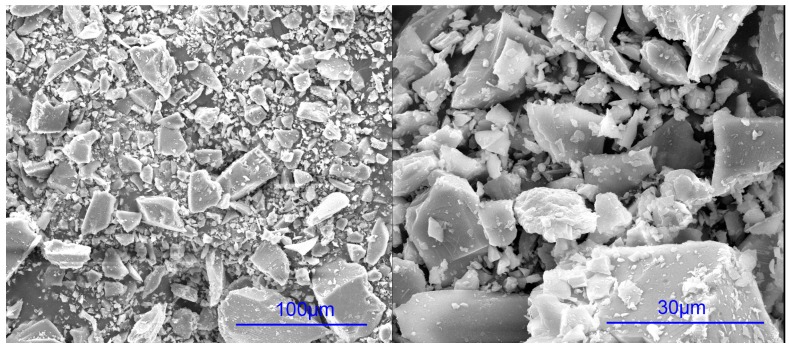
Scanning electron microscope (SEM) pictures of raw glass powder.

**Figure 7:**
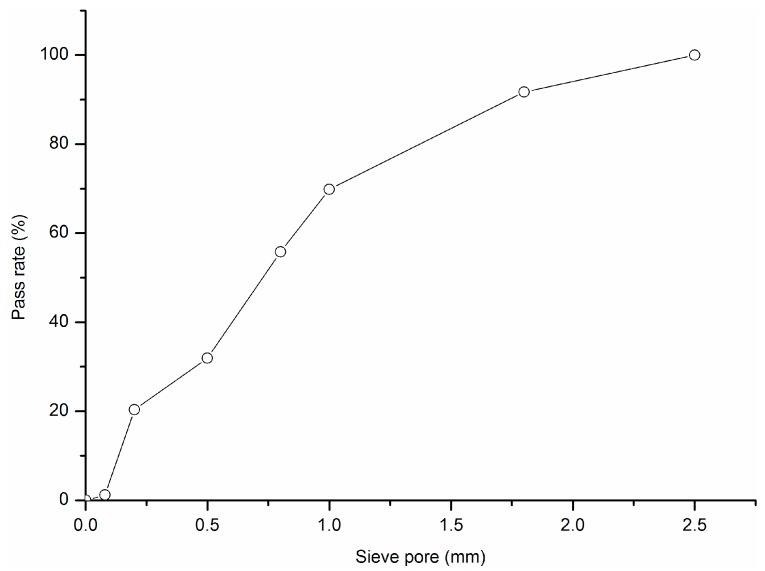
Aggregate grading curve.

The P.I 42.5 cement (China United Cement Corp., Beijing, China) and glass powder from a milled waste glass bottle were used. The waste glass bottle was from wastes disposal station in Shanghai. The chemical composition and particle size distribution of P.I 42.5 cement and the glass powder were tested by X-ray fluorescence spectrometry with SRS 3400 (Bruker AXS, Karlsruhe, Germany) and Laser particle size analyzer LS230 (Beckman Coulter, Indianapolis, IN, USA), respectively. The results are listed in Table 2 and Table 3, respectively. In this study, the waste glass powder had higher SiO_2_, CaO, and Na_2_O, but much lower Al_2_O_3_ contents. According to the Standard Specification for Coal Fly Ash and Raw or Calcined Natural Pozzolan for Use in Concrete (American Society for Testing and Materials, C618) and the chemical composition requirement, the glass powder can be classified as Class N natural pozzolan if Na_2_O content is not a concern. It has been shown that glass powder with particles smaller than 300 μm can effectively mitigate ASR and can even be used as an ASR inhibitor in glass aggregate mortars [44]. D10, D50, and D90 in Table 3 represent the diameters corresponding to 10%, 50%, and 90% in the cumulative particle size distribution curve. In this study, the characteristic particle diameter of D90 was 110 μm, which means 90% of the glass powder is samller than 110 μm. This is better for mitigation of the adverse effects caused by ASR. The X-ray powder diffraction (XRD)analysis of glass powder was carried out by using a Rigaku D/max 2550 X-ray diffractometer (Rigaku Corp., Tokyo, Japan) with Cu Kα radiation generated at 40 kV and 100 mA. The scanning rate was 5°/min. The result is shown in Figure 5. It can be seen that the glass powder mainly consists of amorphous phase. The morphology of glass powder is shown in Figure 6. Silica sand (Xiamen ISO Standard Sand Co., Ltd., Xiamen, China) was used as fine aggregate. The aggregate grading curve is shown in Figure 7. The mortar prepared with pure cement was considered as control group. The replacement ratio of cement by glass powder was 15%, 30% and 45% by weight. The water to binder ratio of mortar was 0.45 by weight. The volume percentage of fine aggregate was 65%. In general, the thickness of ITZ was ~20–50 μm [45]. According to the calculation method mentioned by Sun et al. [46], the ITZ took a great proportion of the paste or even penetrated the paste. The specimens with the diameter of 4 cm and the height of 8 cm were prepared by cylindrical molds processed by Nylon with wall thickness of 4 mm.

Four curing regimes were used in this study. The normal curing was at 20 ± 2 °C with relative humidity (RH) > 90%. The steam curing at 40 °C for 10 h or 80 °C for 4 h had a temperature increase and decrease rate of 15 °C/h. According to the authors’ work [8], the total microwave radiation time of 45 min was selected. Eight specimens with molds on a turntable at one-time were heated up in a microwave oven (Midea Group. Ltd., Shanghai, China) with an output power of 260 W and frequency of 2450 MHz. The specimens were radiated six times in the microwave oven. The specific microwave curing regime was as follows: The interval time between two radiations was 30 min; the first five radiations were 5 min and the last radiation was 20 min.

**Figure 8:**
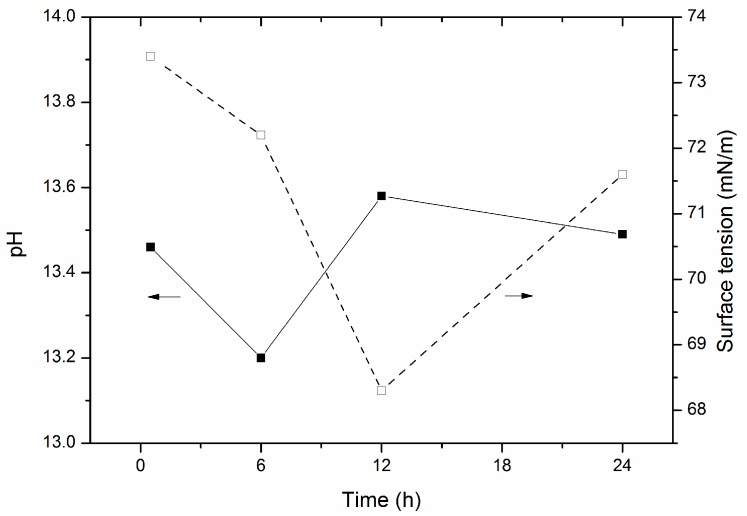
The surface tension and pH of cement-glass powder solution.

Composite binder containing 30% glass powder was used for surface tension and pH test. In this study, 35 g cement and 15 g glass powder with 150 g deionized water (pH = 7.49, Surface tension = 73 mN/m) were stirred rapidly for 5 min in a plastic cup. After curing, the upper solution was tested by a HM 200 pH-meter (HM Digital Inc., Seoul, Korea) and a QBZY-1 automatic surface tension tester (Shanghaihengping Co., Shanghai, China). The results are shown in Figure 8.

The compressive strength of mortar was tested by a hydraulic compression testing machine (Wuxiailikang Co., Wuxi, China) with a loading rate of 2.4 kN/s. For later use in SEM analysis, the cores of broken mortar prepared with composite binder containing 30% glass powder were collected and packed into the ampere bottles filled with absolute ethyl alcohol to terminate hydration. FEI QUANTA 650 and 200 (FEI Co., Hillsboro, OR, USA) were used for the SEM test at one day and 28 days, respectively.

## 5. Conclusions

The improvement of the compressive strength of mortar prepared with composite binder containing glass powder under microwave curing can be attributed to the amelioration of the microstructure of the interfacial transition zone. The effects of microwave curing on the microstructure of the interfacial transition zone can be concluded as follows: The hydration degree of cement is accelerated by the thermal effect of microwave curing and the partially dissolved Na from the fine glass powder to form more reticular C-S-H. This connects the aggregate, calcium hydroxide, non-hydrated cement and glass powder into a denser integral structure, and a more stable triangular structure of calcium hydroxide around the aggregate is formed, rather than a lamellar structure, which contributes to the compressive strength improvement. 

## Figures and Tables

**Table 1:** Element composition of calcium silicate hydrate.

EDS Spots in Figure 4	O	Na	Mg	Al	Si	S	K	Ca	Na/Si	Ca/(Si+Na)
1	62.75	2.29	1.67	2.76	12.87	1.62	0.99	15.05	0.18	0.99
2	59.73	2.82	0.66	1.72	13.62	1.66	0.8	19.01	0.21	1.16
3	44.38	3.09	1.36	2.14	17.12	1.99	1.29	28.62	0.18	1.42
4	34.05	5.07	1.49	3.26	17.22	3.54	2.1	33.27	0.29	1.49

**Table 2:** Chemical composition of P.I 42.5 cement and glass powder (wt %).

	SiO_2_	Al_2_O_3_	Fe_2_O_3_	CaO	MgO	SO_3_	Na_2_O	Cl	f-CaO	Loss on Ignition
Cement	20.81	4.92	3.41	62.65	2.38	2.65	0.67	0.012	0.81	2.01
Glass powder	71.8	1.6	0.39	10.7	0.43	0.46	13.2	0.11	-	0.27

**Table 3:** Particle size distribution and specific surface area of cement and glass powder.

	Cumulative Percentage (%)			Characteristic Particle Diameter (μm)			Specific Surface Area (m^2^/kg)
	<25 μm	<45 μm	<80 μm	D10	D50	D90	
Cement	66.3	89.6	98.4	2.92	17.18	47.94	347
Glass powder	42.7	63.1	81.1	1.75	33.01	110.98	270
